# Solid pseudopapillary neoplasm of the pancreas: a retrospective study of 195 cases

**DOI:** 10.3389/fonc.2024.1349282

**Published:** 2024-02-26

**Authors:** Chang Fu, Xiaocong Li, Yongxin Wang, Chuangshi Wang, Hengwei Jin, Kai Liu, Hongji Xu

**Affiliations:** ^1^ Department of Hepatobiliary and Pancreatic Surgery, General Surgery Center, The First Hospital of Jilin University, Changchun, Jilin, China; ^2^ Medical Research and Biometrics Center, National Clinical Research Center for Cardiovascular Diseases, State Key Laboratory of Cardiovascular Disease, Fuwai Hospital, National Center for Cardiovascular Diseases, Chinese Academy of Medical Sciences and Peking Union Medical College, Beijing, China; ^3^ Department of Abdominal Surgery, Guiqian International General Hospital, Guiyang, Guizhou, China

**Keywords:** solid pseudopapillary neoplasm, pancreatic tumor, surgical treatment, prognosis, risk factors

## Abstract

**Objective:**

Solid pseudopapillary neoplasm of the pancreas (SPN) is a rare exocrine tumor of the pancreas. The aim of our study is to summarize the clinical features of SPN and to analyze the risk factors for malignant SPN.

**Methods:**

From May 2013 to September 2022, patients who were pathologically confirmed to have SPN were retrospectively reviewed. Demographic data, clinical and pathological features, follow-up data were collected and analyzed. To investigate the factors influencing the benign or malignant nature of SPN, we employed logistic regression. Additionally, we utilized Kaplan-Meier curves to depict and analyze the overall prognosis.

**Results:**

A total of 195 patients were included, 163 of whom were female and the average age of all patients was 31.7 years old. Among 195 patients, 101 patients (51.8%) had no obvious clinical symptoms and their pancreatic lesions were detected during routine examination. The primary symptom was abdominal pain and distension in 64 cases (32.8%). The maximum diameter of SPN tumors ranged from 1-17 cm (mean 6.19 cm). Forty-eight postoperative complications developed in 43 (22.1%) patients. After a median follow-up duration of 44.5 months, the overall 5-year survival rate was 98.8% and the recurrence rate was 1.5%. Furthermore, we observed a statistically significant difference in the completeness of the tumor capsule between benign and malignant SPN.

**Conclusion:**

SPN is associated with a favorable long-term survival after surgery in our large sample size cohort. For malignant SPN, tumor capsule incompleteness is an independent risk factor.

## 1 Introduction

Solid pseudopapillary neoplasm of the pancreas (SPN) is a rare tumor, classified as a low-grade malignancy, comprising approximately 0.2% to 2.7% of pancreatic tumors (1). SPN was first reported by Frantz in 1959 (2). In 1996, the World Health Organization (WHO) classified it as a junctional malignant tumor with uncertain biological behavior (3). In 2010, the WHO redefined SPN as a low-grade malignancy and provided specific criteria for its malignancy, which include peripancreatic or deep tissue invasion, vascular invasion, peripheral nerve invasion, distant metastasis, and tumor recurrence (4). SPN predominantly afflicts young women and frequently presents with nonspecific clinical symptoms (5). The preferred treatment for SPN is surgical resection, and the prognosis after SPN is favorable, with a 5-year survival rate of greater than 95%. In recent years, with the improvement of imaging and awareness of SPN, the incidence of SPN has increased significantly, and the number of SPN patients since 2000 is seven times higher than before (6). What’s more, there has been a growing amount of research on SPN lately (7–13). Nonetheless, large cohort with long-term follow-up studies on SPN remain limited, and further research is essential to better understand its clinical features and the risk factors. In this study, we summarized the clinical characteristics, treatment, pathological features, and prognosis of 195 patients with SPN. Furthermore, we conducted an in-depth analysis of the factors contributing to the benign and malignant nature of SPN, aiming to improve the management of this condition.

## 2 Materials and methods

This retrospective study encompassed 195 SPN patients who underwent surgery at our hospital between May 2013 and September 2022. We systematically gathered and analyzed comprehensive clinical data, pathological features, and prognosis. The study was approved by First Hospital of Jilin University Research Ethics Committee, and the requirement for informed consent was waived due to the retrospective study design.

### 2.1 Data collection

We collected demographic data and clinical data from our hospital’s electronic medical records. Demographic data including sex, age and body mass index (BMI). Clinical data including chief complaint, preoperative liver function, preoperative tumor marker, imaging findings, surgical procedures and postoperative complications. Pathologic review was performed retrospectively by two pathologists. Pathological features including tumor size, margin status, Ki-67 index, tumor components and immunohistochemical results. The severity of postoperative complications was classified according to Clavien-Dindo Classification (14). Severe complications were defined as Clavien-Dindo grade III or greater. Complications were defined according to the International Study Group of Pancreatic Surgery (ISGPS), which include postoperative pancreatic fistula (POPF), delayed gastric emptying (DGE), and postoperative hemorrhage (POPH) (15–17). Survival time and prognosis were collected through follow-up visits.

### 2.2 Follow-up

All participants were followed up through clinic visits or telephone communication. The starting point for follow-up was set at postoperative day 1, and the end point for follow-up was November 30, 2022 or death of the patient. Endpoint events were tumor recurrence, metastasis, or death. Specifically, we defined overall survival (OS) as the duration between the surgical procedure and either the patient’s passing or the final follow-up visit. For recurrence-free survival (RFS), we measured the time from the surgical intervention to the occurrence of tumor recurrence. Recurrence was defined as a local or a metastatic tumor confirmed by radiology or histology during postoperative follow-up.

### 2.3 Statistics

The clinical characteristics were described using mean ± standard deviation or median (Interquartile Range (IQR)) for quantitative indicators; and number of cases (percentage) for qualitative indicators. Follow-up was described using the median follow-up time, and survival analysis was performed using the Kaplan-Meier method. Characteristics of the study subjects were reported by benign-malignant subgroups, and between-group differences were tested using Student t test and Wilcoxon test for continuous parameters, chi-square test and Fisher’s exact test for categorical parameters. To investigate the risk factors associated with the benign or malignant nature of SPN, we conducted multifactorial analysis through logistic regression. All statistical analyses were performed using SPSS 24.0 (IBM SPSS Statistics, Armonk, NY). P<0.05 was considered statistically significant difference.

## 3 Results

### 3.1 Patients

**Figure 1:**
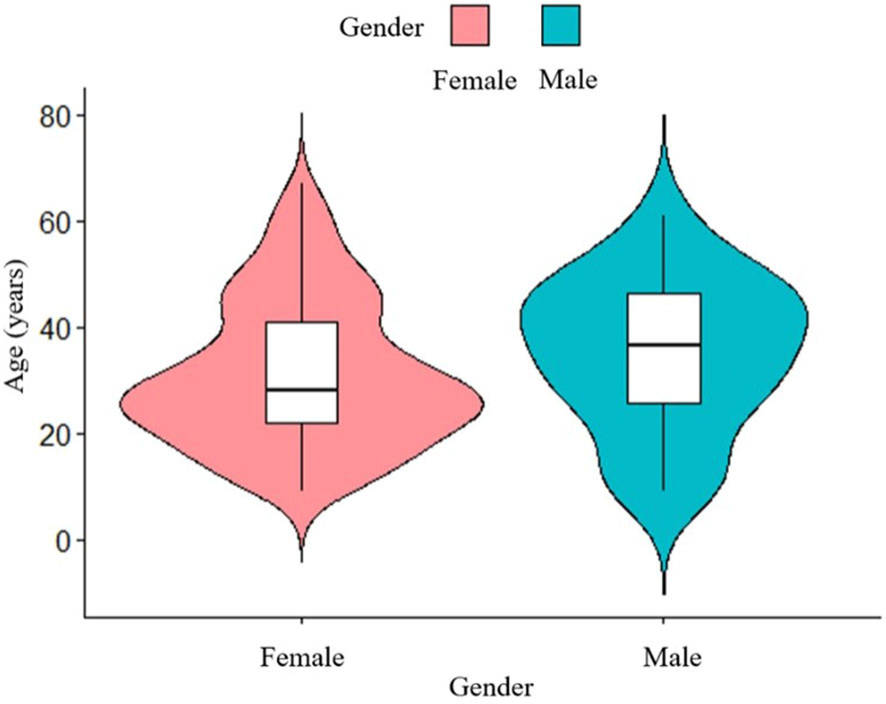
Age-frequency distribution of male and female SPN patients. The peak age of SPN onset is different between male and female.

**Figure 2:**
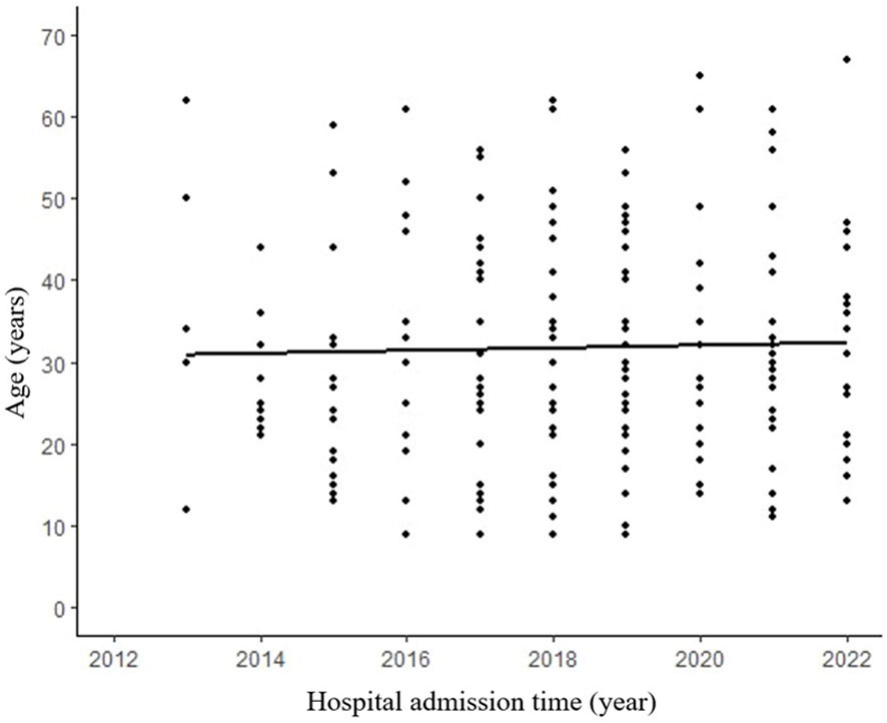
No significant difference between admission time and age. R² refers to the goodness of fit of the regression line, indicating the degree to which the regression line fits the observed values. (R²=0.004, p=0.688).

A total of 195 patients with an age range of 9-67 years (median age 27 years) were included in this study, with a mean age of 35.1 ± 14.1 years for male patients and 31.1 ± 13.7 years for female patients. The study included 163 female patients and 32 male patients, with a male to female ratio of 1:5.1. Analysis of age-frequency distribution revealed a unimodal skewed pattern for SPN in male patients, peaking around 40-50 years of age. In contrast, female patients exhibited a bimodal distribution, with early onset peaking at 20-30 years and late onset peaking at 40-50 years (**Figure 1**). Analysis of the relationship between the age and the time of admission to the hospital showed that the age of the patients was about 30 years old in the last 10 years, which did not change significantly (**Figure 2**). 101 patients (51.8%) had no obvious clinical symptoms and their pancreatic lesions were detected during routine examination using ultrasonography (US) or computed tomography (CT). The remaining patients presented with abdominal pain and distension in 64 cases (32.8%), abdominal discomfort in 8 cases (4.1%), abdominal mass found in 6 cases (3.1%), nausea and vomiting in 5 cases (2.6%), back pain in 4 cases (2.1%), vomiting of blood in 3 cases (1.5%), jaundice in 2 cases (1.0%), and diarrhea in 2 cases (1.0%).

### 3.2 Laboratory and imaging examinations

Elevated tumor markers were infrequently observed in SPN, with 5.6% showing increased levels of neuron specific enolase (NSE), followed by carcinoembryonic antigen (CEA) (2.2%), carbohydrate antigen 19-9 (CA 19-9) (1.1%), and alpha fetoprotein (AFP) (0.5%). The most commonly employed diagnostic modalities were CT and magnetic resonance imaging (MRI). SPN demonstrated a diverse distribution throughout the pancreas, with 125 (64.1%) located in the body or tail, 68 (34.9%) in the head or neck. Furthermore, two cases presented with multiple tumors—one involving the head and body of the pancreas, and the other involving the head and tail. The tumor components varied, with cystic in 30 cases, solid in 79 cases, and cystic-solid in 86 cases. Calcifications were observed in 84 cases. Enhanced CT was performed in 185 patients preoperatively, of which 139 were diagnosed with SPN, with a diagnostic accuracy of 72.8%.

### 3.3 Surgical procedures and postoperative complications

All patients underwent surgical treatment, with 129 patients (66.2%) underwent laparoscopic surgery and 66 patients (33.8%) underwent open surgery. The surgical procedures included spleen-preserving distal pancreatectomy (n=76), distal pancreatectomy with splenectomy (n=57), pancreaticoduodenectomy (n=35), local resection (n=17), central pancreatectomy (n=5), pylorus-preserving pancreaticoduodenectomy (n=4), total pancreatectomy (n=1). Additionally, three patients underwent combined partial hepatectomy because of liver metastasis. Importantly, R0 resection, indicating complete tumor removal, was achieved for all patients.

**Figure 3:**
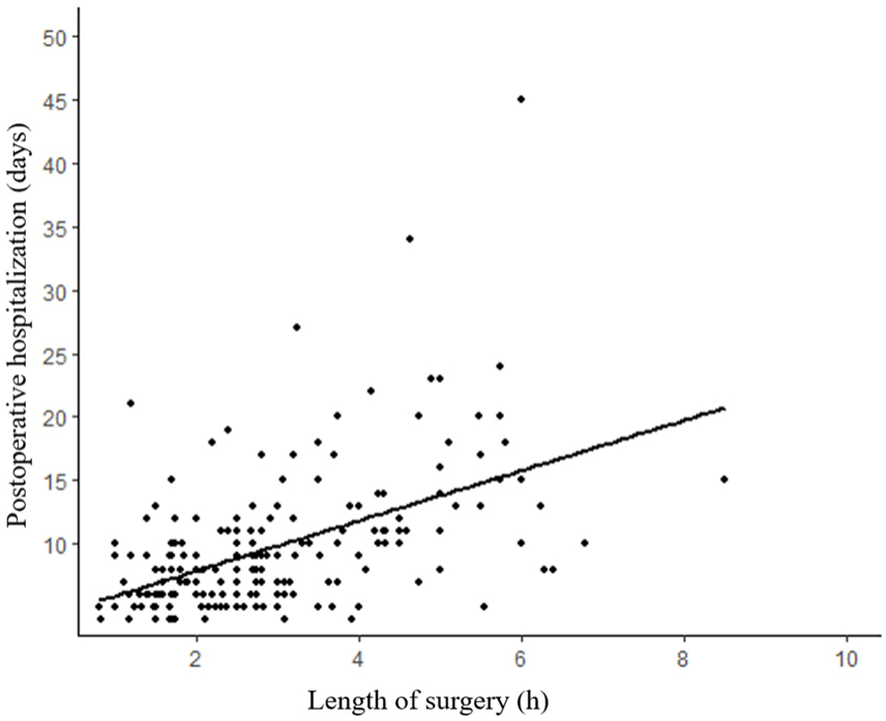
Length of postoperative hospitalization tends to increase with duration of surgery. (R²=0.260, p<0.001).

**Figure 4:**
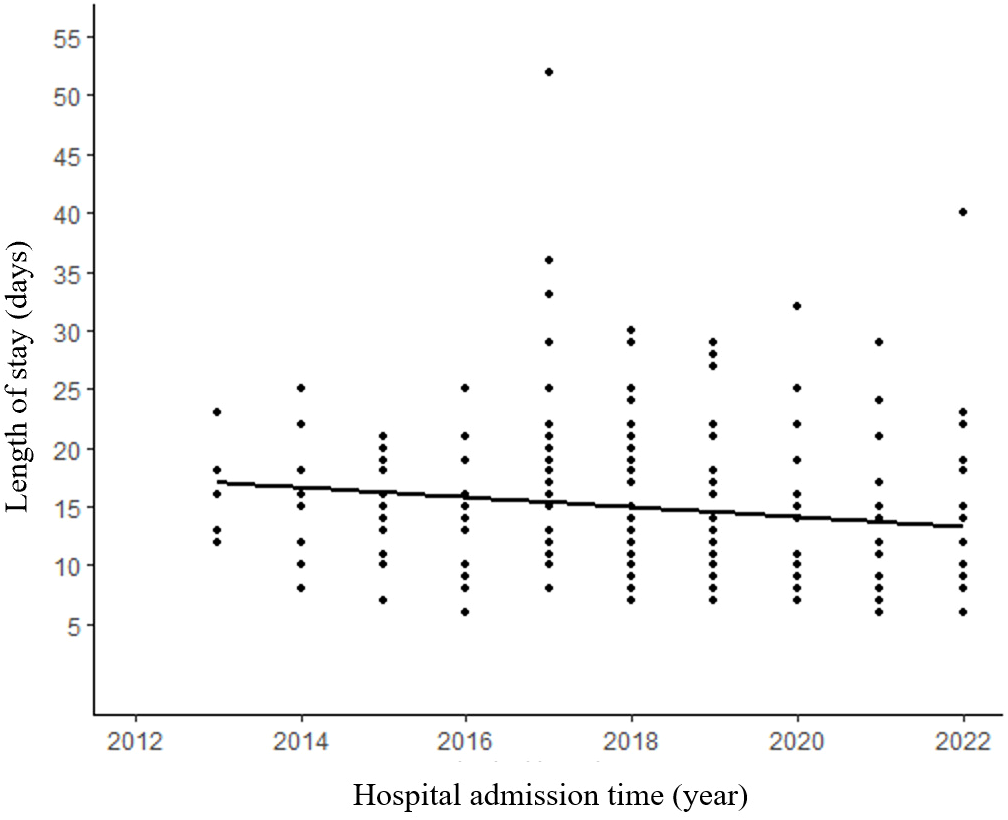
Patients in recent years tend to have shorter length of hospitalization. (R²=0.018, p=0.036).

The median surgical duration was 2.7 (IQR: 1.8–3.8) hours. An analysis of the correlation between the length of surgery and postoperative hospitalization revealed a noteworthy upward trend with increasing surgical duration (**Figure 3**). The median length of stay for all patients was 8 days (IQR:6-11 days). Furthermore, our analysis of the relationship between the admission time and the length of stay demonstrated a significant decrease in the duration of hospitalization (p=0.036) (**Figure 4**).

Forty-eight postoperative complications developed in 43 (22.1%) patients. The most common postoperative complication is POPF, 20 patients were type A or type B fistula, 23 patients developed type C fistula. A female patient developed perioperative mortality due to severe postoperative pancreatic fistula. Two patients developed DGE and were treated conservatively. Three patients developed POPH and required re-operation.

### 3.4 Pathologic and immunohistochemical characteristics

**Figure 5:**
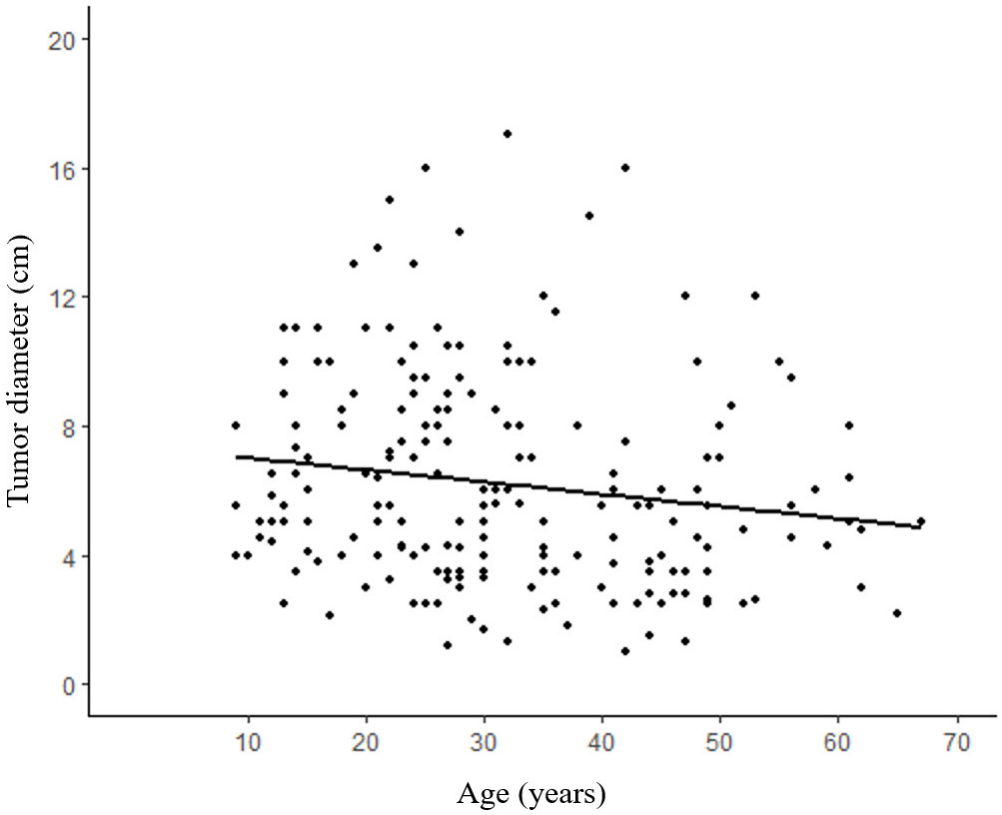
Correlation between age and tumor size. Older patients tend to have relatively smaller SPN compared with young patients. (R²=0.020, p=0.026).

The maximum diameter of the tumors ranged from 1-17 cm (media:5.5 cm), and the mean diameter of the tumors was 5.31 ± 3.20 cm in male patients and 6.36 ± 3.25 cm in female patients. A correlation analysis between tumor diameter and patient age revealed a significant association, indicating that younger patients tended to have larger tumors (p=0.026) (**Figure 5**).

Tissue invasion was reported in 32 patients, followed by perineural invasion (n=25), nearby organs invasion (n=8), distant metastasis (n=2). A tumor capsule was present in the majority of cases, with 179 patients (91.8%) exhibiting this characteristic.

Immunohistochemically, almost all the tumors were strongly positive for alpha 1-antichymotrypsin (AACT) 97.4%, vimentin (Vim) 100%, CD10 96.8%, CD56 100%, Cyclin-D1 95.3%, β-catenin 99.4%, progesterone receptor (PR) 98.5%, synaptophysin (Syn) 92.4%. Notably, chromogranin A (CgA) showed negative expression in 97.3% of cases (**Table 1**). The median Ki-67 index was 1.0% (range 1%-10%).

**Table 1:** Results of immunohistochemical examination.

Antigen	Number	Positive %
Vimentin	184	100%
AACT	114	97.40%
CK-pan	165	61.80%
CD10	185	96.80%
CD56	167	100%
Cyclin-D1	43	95.30%
β-Catenin	170	99.40%
PR	132	98.50%
CgA	187	2.70%
Syn	184	92.40%

### 3.5 Follow-up results

One patient died from complications during the perioperative period, 24 patients were lost to follow-up, and the other patients were followed up until November 2022. The median follow-up duration was 44.5 months (range, 2–115 months). During the follow-up period, three female patients developed tumor recurrence, of which two patients died and one patient underwent surgery with a good prognosis (**Table 2**). Patient 1 was followed up annually after three years of regular semiannual follow-up. Re-examination revealed an unresectable liver metastasis. We suggested radiofrequency ablation or chemotherapy to the patient, but the patient refused. Patient 3 developed a severe pancreatic fistula (type C) after spleen-preserving distal pancreatectomy and was readmitted for conservative management after discharge. Patient 3 was unable to undergo surgical resection of liver metastasis due to poor nutritional status, and chemotherapy was terminated due to physical intolerance. The recurrence rate was 1.5%.

**Table 2:** Clinicopathological data of the recurrent patients.

Patient	Age, Yr	Gender	BMI	Chief complaint	Tumor location	Metastasis before surgery	Surgical approach	Surgical procedure	Tumor invasion	Tumor component
Patient 1	59	Female	22.5	Abdominal distension	Body-Tail	NO	Laparoscopic	SPDP	None	Cystic-Solid
Patient 2	9	Female	21.4	Abdominal pain	Body-Tail	NO	Laparoscopic	SPDP	Peripancreatic invasion	Cystic-Solid
Patient 3	62	Female	23.4	Abdominal pain	Body-Tail	NO	Laparoscopic	SPDP	None	Cystic

**Table:** 

Patient	Tumor size, cm	Tumor calcification	Tumor capsule	Ki-67	Recurrence location	Single or multiple metastasis	Treatment for recurrence	OS, months	RFS, months	Current status
Patient 1	4.3	Calcified	Complete	2	Liver	Single	Follow-up	52	52	Dead
Patient 2	5.5	Non-calcified	Incomplete	5	Adrenal and peritoneal	Multiple	Surgery	81	50	Alive
Patient 3	4.8	Calcified	Complete	1	Liver	Single	Follow-up	4	4	Dead

SPDP, Spleen-preserving distal pancreatectomy.

**Figure 6:**
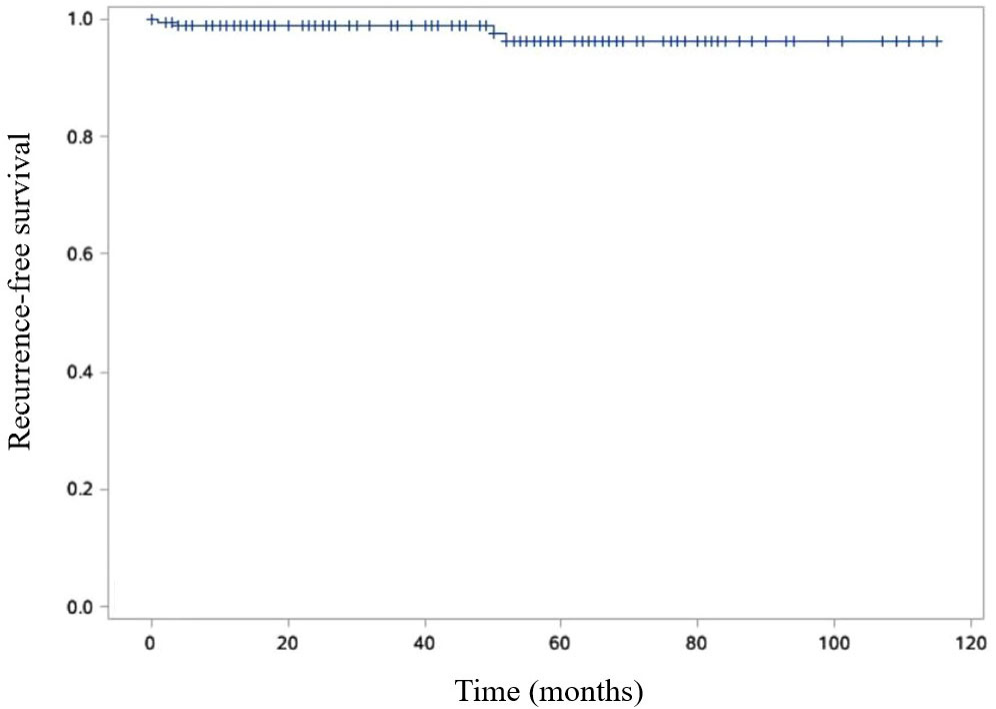
Kaplan-Meier curve is shown RFS of SPN patients. Recurrence free survival (RFS) was defined as the time from the surgical intervention to the tumor recurrence.

**Figure 7:**
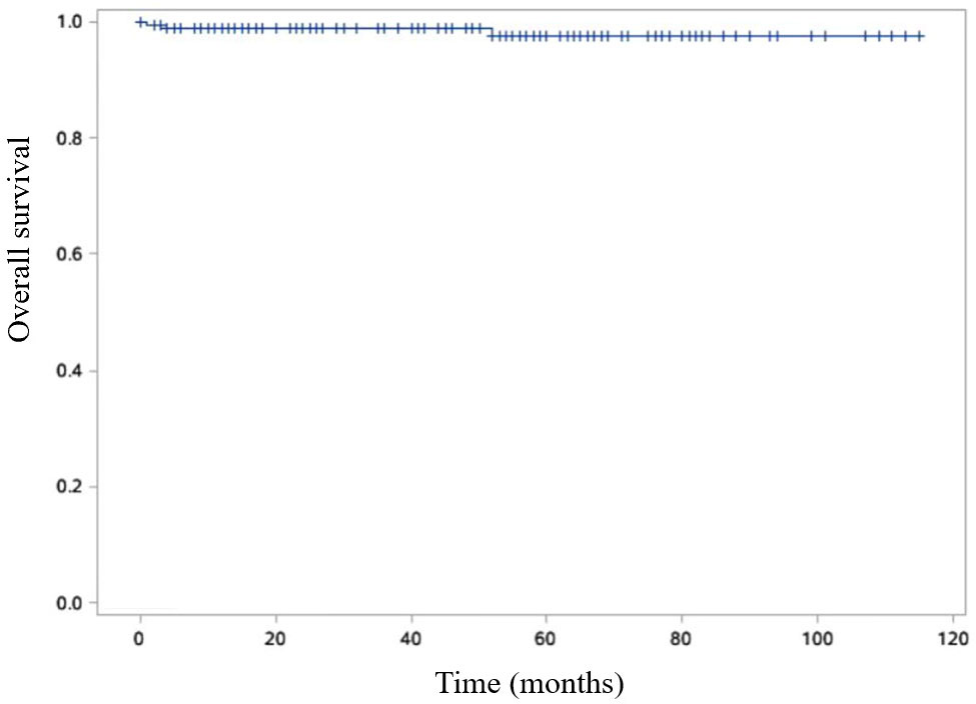
Kaplan-Meier curve is shown OS of SPN patients. Overall survival (OS) was defined as the duration between the surgical procedure and either the patient’s passing or the final follow-up visit.

The disease-free survival rates at 3- and 5- years post-surgery were impressively high at 99.4% and 98.2%, respectively (**Figure 6**). Additionally, the 3- and 5-year survival rates were equally remarkable at 99.4% and 98.8%, respectively (**Figure 7**).

### 3.6 Comparison of general characteristics and risk factors in SPN with different malignant potential

According to the 2010 WHO classification for benign and malignant SPN, patients were categorized into benign and malignant groups (4). Among them, 140 cases were identified as benign SPN, and 55 cases were classified as malignant SPN. The parameters, including age, gender, BMI, duration of surgery, duration of hospitalization, tumor capsule, tumor size, tumor components, tumor calcification and Ki-67 index were analyzed between two groups. There was a statistically significant difference in tumor capsule integrity between benign and malignant SPN(p=0.043). However, other parameters were not statistically significant.

For OS, the benign group had a median survival time of 42.0 (IQR:16.0–63.5) months, while the malignant group had a median OS of 24.0 (IQR:9.0–65.0) months. Although there was a noticeable difference in OS between the two groups, it did not reach statistical significance (p=0.137). Regarding RFS, the benign group exhibited a median RFS of 42.0 (IQR:16.0–63.5) months, while the malignant group had an RFS of 24.0 (9.0–59.0) months. While a difference in RFS between the benign and malignant groups was observed, it was not statistically significant (p=0.106) (**Table 3**).

**Table 3:** Characteristics of patients with SPN according to the 2010 WHO classification.

Variables	Total (N=195)	Benign (N=140)	Malignant (N=55)	P value
**Age, Yr**				0.783
Median (IQR)	29.0 (22.0–43.0)	29.5 (23.0–42.0)	28.0 (19.0–44.0)	
**Gender**				0.676
Male	32 (16.4)	22 (15.7)	10 (18.2)	
Female	163 (83.6)	118 (84.3)	45 (81.8)	
**BMI**				0.122
Mean ± SD	23.7 ± 4.2	24.0 ± 4.4	23.0 ± 3.8	
**Duration of surgery, h**				0.409
Median (IQR)	2.7 (1.8–3.8)	2.5 (1.8–3.8)	2.7 (1.9–4.3)	
**Duration of postoperative hospitalization**				0.913
Median (IQR)	8.0 (6.0–11.0)	8.0 (6.0–11.5)	8.0 (6.0–11.0)	
**Capsule**				0.043
Incomplete	16 (8.2)	8 (5.7)	8 (14.5)	
Complete	179 (91.8)	132 (94.3)	47 (85.5)	
**Tumor size, cm**				0.416
Median (IQR)	5.5 (3.7–8.0)	5.5 (3.5–8.6)	5.5 (3.8–7.0)	
**Tumor components**				0.545
Cystic-Solid	86 (44.1)	61 (43.6)	25 (45.5)	
Cystic	30 (15.4)	24 (17.1)	6 (10.9)	
Solid	79 (40.5)	55 (39.3)	24 (43.6)	
**Tumor calcification**				0.288
Non-calcified	111 (56.9)	83 (59.3)	28 (50.9)	
Calcified	84 (43.1)	57 (40.7)	27 (49.1)	
**Ki-67**				0.055
Median (IQR)	1 (1-2)	1 (1-2)	2 (1-3)	
**OS, month**				0.137
Median (IQR)	40. 0 (13.0–64.0)	42.0 (16.0–63.5)	24.0 (9.0–65.0)	
**RFS, month**				0.106
Median (IQR)	40. 0 (13.0–63.0)	42.0 (16.0–63.5)	24.0 (9.0–59.0)	

The incomplete tumor capsule suggests that the nature of SPN tends to be malignant, aligning with its biological behavior of invasive growth by breaching the tumor capsule. As expected, both OS and RFS in the benign group showed a better prognosis compared to the malignant group.

To explore the factors related to benign and malignant SPN, we conducted multifactorial analysis using a logistic regression risk model, which included age, gender, BMI, tumor size, tumor capsule integrity, calcification, location, and tumor components. The results indicated a statistically significant difference in tumor capsule incompleteness between benign and malignant SPN (p=0.035). While BMI, tumor components, and calcification exhibited differences between benign and malignant SPN, these differences were not statistically significant (**Table 4**).

**Table 4:** Multivariate analysis of factors related to benign and malignant SPN.

Variables		OR (95%CI)	P value
**Age, Yr**		1.002 (0.975-1.029)	0.890
**Gender**	Female	Reference	
	Male	0.900 (0.361-2.244)	0.821
**BMI**		0.939 (0.857-1.029)	0.176
**Tumor size**		0.942 (0.838-1.060)	0.324
**Capsule**	Complete	Reference	
	Incomplete	3.257 (1.084-9.785)	0.035
**Tumor calcification**	Non-calcified	Reference	
	Calcified	1.605 (0.795-3.237)	0.187
**Tumor components**	Cystic-Solid	Reference	
	Cystic	0.472 (0.162-1.373)	0.168
	Solid	0.925 (0.434-1.971)	0.452
**Location**	Head	Reference	
	Body-Tail	1.429 (0.721-2.833)	0.307
**Chief complaint**	Symptomatic	Reference	
	Asymptomatic	0.842 (0.425-1.669)	0.622

OR, Odds Ratio.

## 4 Discussion

SPN is a rare low-grade malignant tumor characterized by solid and pseudopapillary structures (18). Kosmahl et al (19) suggested that SPN derives from genital ridge/ovarian anlage-related cells which were attached to the pancreatic tissue during early embryogenesis. Some studies suggest that the development of SPN is associated with molecular alterations in the Wnt/β-catenin and Notch signaling pathways (20, 21). Furthermore, genetic mutations within exon 3 of the β-catenin gene and the downregulation of E-cadherin signaling are frequently observed in tumor cells (22, 23).

SPN tends to occur in young women, and a study that included 2,744 patients showed that the average age of the patients was 28.5 years, with 87.8% female patients (6). Results from another study that included 2,450 SPN patients in 2020 showed the average age was 29.3 years, with 84.1% being female (24). In alignment with these findings, our study revealed a mean patient age of 31.7 years, with 83.6% of patients being female, further confirming the typical demographic characteristics. The age pattern of SPN onset varies between genders, consistent with previous research by Wu et al (5), who found a bimodal age-frequency distribution among female patients and a unimodal skewness distribution among male patients. This pattern suggests that sex hormones may play a role in the development of SPN (25). Distinctions in clinical characteristics based on gender differences among SPN patients were also found in another study (26). SPN primarily occurs in the body-tail region. A study by Yu et al (27) found that the mean diameter of the tumor was 7.87 cm and the preferred site was the body-tail of the pancreas (54.8%). Similarly, Song et al (28) reported a mean tumor size of 6.4 cm, with 60.4% of tumors occurring in the body-tail region. Our study aligns with these findings, as we identified a mean tumor size of 6.2 cm, with 64.1% of cases located in the body-tail of the pancreas.

SPN typically lacks specific clinical manifestations. About 1/3 of the patients have no obvious clinical symptoms, which are found by routine examination (6). The primary clinical manifestations include abdominal pain, abdominal discomfort, nausea, vomiting, low back pain, and the detection of abdominal masses (24, 29). In our study, 51.8% of patients were asymptomatic, while the most common clinical symptoms were abdominal pain and distension. Jaundice is rarely in SPN patients (12, 30, 31), and our study found only one patient with a tumor located in the head of the pancreas who developed jaundice. Laboratory tests are usually normal and most tumor markers are negative (24, 32). Tumor markers lack specificity for diagnosing SPN and can be used to differentiate it from pancreatic cancer.

Due to the limited specificity of laboratory tests in diagnosing for SPN, imaging techniques such as CT, MRI, and US are important for its diagnosis (33). With the advancement of imaging techniques, the accuracy of preoperative diagnosis for SPN has further increased. In the first five years of our study, the preoperative imaging accuracy rate for SPN was 68.5%, which increased to 76.4% in the last four years. CT is the most frequently used imaging modality for preoperative diagnosis of SPN (6, 34). In this study, 185 patients underwent preoperative abdominal enhanced CT, with 139 of them receiving a confirmed SPN diagnosis, resulting in a diagnostic accuracy rate of 72.8%. Compared with CT, MRI has higher tissue resolution and can better present the relationship between the tumor and the bile ducts and pancreatic ducts, which is clinically important for planning surgical approaches (35). Endoscopic Ultrasound Guide-Fine Needle Aspiration Biopsy (EUS-FNA) is an important means of obtaining preoperative tumor tissue samples, with some studies reporting an accuracy rate of over 80% for preoperative SPN diagnosis using this technique (36). However, considering the fact that EUS-FNA is an invasive test, as well as the possible risks of bleeding, infection, and tumor needle tract dissemination, its application has not yet been widely conducted.

Surgical resection remains the preferred treatment for SPN (37). The choice of surgical procedure needs to be based on tumor size, location and rapid intraoperative pathological diagnosis. Some studies have suggested that surgical resection should be performed even if preoperative invasion of peripheral organs or distant metastasis have been detected (27, 38, 39). Due to the low malignancy of SPN, tumor resection with preservation of pancreatic function should be performed as much as possible while ensuring negative margins. Pylorus preserving pancreaticoduodenectomy, spleen-preserving distal pancreatectomy, central pancreatectomy, tumor enucleation, duodenum-preserving pancreatic head resection, and so on, seem reasonable procedures for the treatment of SPN (1, 40). Given the generally indolent nature of SPN and its low risk of lymph node metastasis, routine lymph node dissection was not performed.

The diagnosis of SPN primarily relies on pathology and immunohistochemistry. Characteristic pathological features of tumor tissues show that it consists of solid, pseudopapillary and cystic areas with typical tumor cells growing around the axis of fibrous blood vessels to form the branching pseudopapillary structure or the pseudodomiform cluster structure (41). SPN has no specific immunophenotype. In most studies, immunohistochemistry results have shown high positive expression of β-Catenin, PR, Syn, AACT, Vim, and CD56 in SPN, while CgA is typically negatively expressed (24, 27, 42, 43). In our study, we observed immunopositivity exceeding 90% for β-Catenin, PR, Syn, AACT, Vim, CD10, CD56, and Cyclin-D1, with a CgA negative expression rate of 97.3%. Immunohistochemical features play a crucial role in distinguishing SPN from pancreatic cancer and neuroendocrine tumors (44, 45). Several studies recommend applying a combination of immunophenotypic markers to improve diagnostic accuracy (45, 46). Ki-67 is an indicator of tumor cell proliferation. Given SPN’s low-grade malignant nature and relatively indolent behavior, Ki-67 is often low. A study has indicated that Ki-67 ≥4% is associated with a poor prognosis (47). In our study, Ki-67 was higher in the malignant group than in the benign group, but the difference was not statistically significant.

Due to the low malignancy and favorable prognosis of SPN, there are still no definite conclusions regarding its malignancy and risk factors for recurrence and metastasis. Various studies have explored the relationship between SPN characteristics and malignancy. For instance, Kang et al (48) proposed that tumor larger than 5 cm may indicate SPN with malignant potential, while Lubezky et al (43) suggested a correlation between larger tumor sizes and metastasis. However, Robertis et al (49) reported that tumor size was not related to tumor malignancy. In this study, incomplete tumor capsule was found to be an independent risk factor for malignant SPN, while factors like tumor size, calcification, and component did not exhibit statistically different between benign SPN and malignant SPN. Consider that possibly due to the incompleteness of the tumor capsule, it can be more closely related to the surrounding tissues to the extent that invasion of peripheral blood vessels, nerves, and peripancreatic adipose tissue is more likely to occur. Yang et al. found that tumor size and Ki-67 were independent predictors of RFS, and based on this, they constructed the Fudan Prognostic Index (29). This grading system showed a significant difference in RFS for the low, intermediate, and high-risk groups of SPN, but no significant difference in OS for the low-risk and intermediate-risk groups. Chen et al (50) showed that tumor size, lymphovascular infiltration, and Ki-67 were independent risk factors for SPN recurrence. Additionally, the synthesis of relevant research can help shape personalized follow-up strategies for patients within each risk category, ultimately facilitating early detection and treatment of recurrence and metastasis in high-risk individuals.

SPN often has a favorable prognosis after surgical resection. In a meta-analysis involving 718 patients with SPN, the 5-year survival rate was 95% (30). Another study conducted by Liu et al (51), which analyzed 243 SPN patients with a median follow-up of 46 months, reported an exceptional 5-year survival rate of 98.4%. Even if recurrence or metastasis occurs after surgery, long survival is often achievable through reoperation. In this study, the median follow-up period was 44.5 months, ranging from 2 to 115 months. During the follow-up, we observed three cases of tumor recurrence and metastasis, resulting in two deaths. One patient experienced peritoneal and adrenal metastasis, and the disease-free survival is 31 months following reoperation. Our study demonstrated 3-year and 5-year survival rates of 99.4% and 98.8% after surgery. This shows that patients with SPN can have a good prognosis through surgery. Due to the limited number of patients with SPN recurrence and the lack of postoperative pathological data of metastatic tumors, more cases need to be studied in the future.

This retrospective study has several limitations. First, there is no standardized criterion for differentiating cystic, solid and calcified components of tumors, and this may introduce subjectivity when compared to other studies. Second, due to the long follow-up period, variations in tumor immunohistochemistry standards and methodologies might exist, leading to deficiencies in the analysis of specific expression or high positive rate expression in immunohistochemical phenotyping. Finally, the prognosis of this disease is good, even if long-term follow-up has been carried out, the number of patients with recurrence, metastasis or death after surgery remains relatively low. This limitation affects our ability to conduct an in-depth analysis of the factors influencing prognosis. Future investigations with larger sample sizes and long-term follow-up periods will be essential to provide a more comprehensive understanding of these factors.

## 5 Conclusions

In conclusion, as a low-grade malignant pancreatic tumor, SPN is most frequently found in young women aged 20-30 years old. It mostly located in the body-tail region of the pancreas. SPN often has no obvious clinical manifestations. Laboratory tests are mostly normal, and preoperative diagnosis is based on CT imaging, while confirmation of diagnosis is based on pathological and immunohistochemical results. Incomplete tumor capsule is identified as an independent risk factor for malignant SPN. The preferred treatment for SPN is surgical resection, which typically results in a favorable postoperative prognosis.

## Data availability statement

The raw data supporting the conclusions of this article will be made available by the authors, without undue reservation.

## Ethics statement

The studies involving humans were approved by First Hospital of Jilin University Research Ethics Committee. The studies were conducted in accordance with the local legislation and institutional requirements. Written informed consent for participation was not required from the participants or the participants’ legal guardians/next of kin in accordance with the national legislation and institutional requirements.

## Author contributions

CF: Conceptualization, Data curation, Writing – original draft. XL: Formal analysis, Methodology, Writing – original draft. YW: Validation, Visualization, Writing – original draft. CW: Formal analysis, Methodology, Supervision, Writing – review & editing. HJ: Data curation, Software, Validation, Writing – review & editing. KL: Conceptualization, Project administration, Writing – review & editing. HX: Investigation, Project administration, Supervision, Writing – review & editing.
